# UHPLC-Q-TOF-MS/MS and Network Pharmacology Analysis to Reveal Quality Markers of Xinjiang *Cydonia oblonga* Mill. for Antiatherosclerosis

**DOI:** 10.1155/2022/4176235

**Published:** 2022-05-28

**Authors:** Jimilihan Simayi, Abulaiti Abulizi, Maimaitiming Nuermaimaiti, Nawaz Khan, Sendaer Hailati, Mengyuan Han, Ziruo Talihati, Kayisaier Abudurousuli, Nulibiya Maihemuti, Muhadaisi Nuer, Wenting Zhou, Ainiwaer Wumaier

**Affiliations:** ^1^Department of Pharmacology, Xinjiang Medical University, 830011 Urumqi, Xinjiang, China; ^2^Institute of Traditional Uyghur Medicine, Xinjiang Medical University, 830011 Urumqi, Xinjiang, China

## Abstract

*Cydonia oblonga* Mill. (COM), mature fruit of genus Rosaceae, is consumed as a kind of traditional Chinese medicinal herb. Previous studies have shown that the components in COM extract have antioxidant, anti-inflammatory, blood pressure-lowering, blood lipid-lowering, antithrombotic, and other biological activities. However, the quality markers (Q-markers) of atherosclerosis (AS) have not been elucidated. The Q-marker is based on the five core principles of traceability, transferability, specificity, measurability, validity, and prescription dispensing. In this study, the quality markers of quince were investigated by applying the ultraperformance liquid chromatography-time-of-flight mass spectrometry (UHPLC/Q-TOF-MS/MS) method and network pharmacology method to highlight the three core elements which are, respectively, traceability transmission, measurability, and validity. At the first step, 72 components were identified by applying the ultraperformance liquid chromatography-time-of-flight mass spectrometry (UHPLC/Q-TOF-MS/MS) method. In the next step, 46 candidate components of COM anti-AS were obtained by network pharmacology, and then, 27 active components were filtered with the molecular docking assay. Finally, the 27 active components were intersected with 10 active components obtained by mass transfer and traceable quality markers. Four anti-AS Q-markers of COM were identified, including caffeic acid, chlorogenic acid, ellagic acid, and vanillic acid, which provided a reference for the quality control of quince. The methods and strategies can also be applied to other traditional Chinese medicines and their compound preparations, providing new ideas on the quantitative evaluation and identification of quality markers.

## 1. Introduction

*Cydonia oblonga* Mill. (COM), a traditional Chinese medicine (TCM) with Xinjiang characteristics, is effective in reducing blood pressure, lowering blood fat, supplementing blood volume and improving brain health, relieving cough, resolving phlegm, and appetizing, as well as relieving diarrhea symptoms, etc. It is mostly used in treating cardiovascular diseases and gastrointestinal diseases and is most efficient in relieving cough and diarrhea [1, 2].

COM is rich in various active components which present potential medical therapeutical value. The domestic research data on the chemical composition analysis of Xinjiang COM is limited. Parida Abuliz et al. demonstrated that Xinjiang COM fruits contain polysaccharides, phenolic acids, flavonoids, alkaloids, tannins, organic acids, volatile oils, triterpenoids, and other components by mutual validation of two methods: systematic pretesting and special pretesting [3]. Our previous studies have shown that COM extract has hypotensive effects, as well as biological activities such as antioxidant, anti-inflammatory, hypolipidemic, hypoglycemic, and antithrombotic [4–7], which are important pathological factors in the pathogenesis and development of atherosclerosis (AS). It is the main pathological basis of cardiovascular diseases, on which plaque growth, rupture, and thrombosis can lead to acute cardiovascular events and seriously endanger human health.

The quality markers (Q-markers) in a TCM are the core concept of quality evaluation and quality control of Chinese medicine, proposed by Academician Liu Chang-xiao, based on the five core principles of traceability and transferability, specificity, measurability, efficacy, and prescription dispensing [8–10]. The Q-marker is a marker representing the characteristics of a certain Chinese medicine, which are of clear origin, related to the therapeutic use, a combination of compounds reflecting the quality characteristics of Chinese medicine, associated with the functional properties of Chinese medicine, revealing the integrity and also effectiveness of Chinese medicine, and providing a scientific basis for quality control of Chinese medicine. Therefore, it is necessary to further define the Q-marker of COM for quality control of COM.

**Figure 1:**
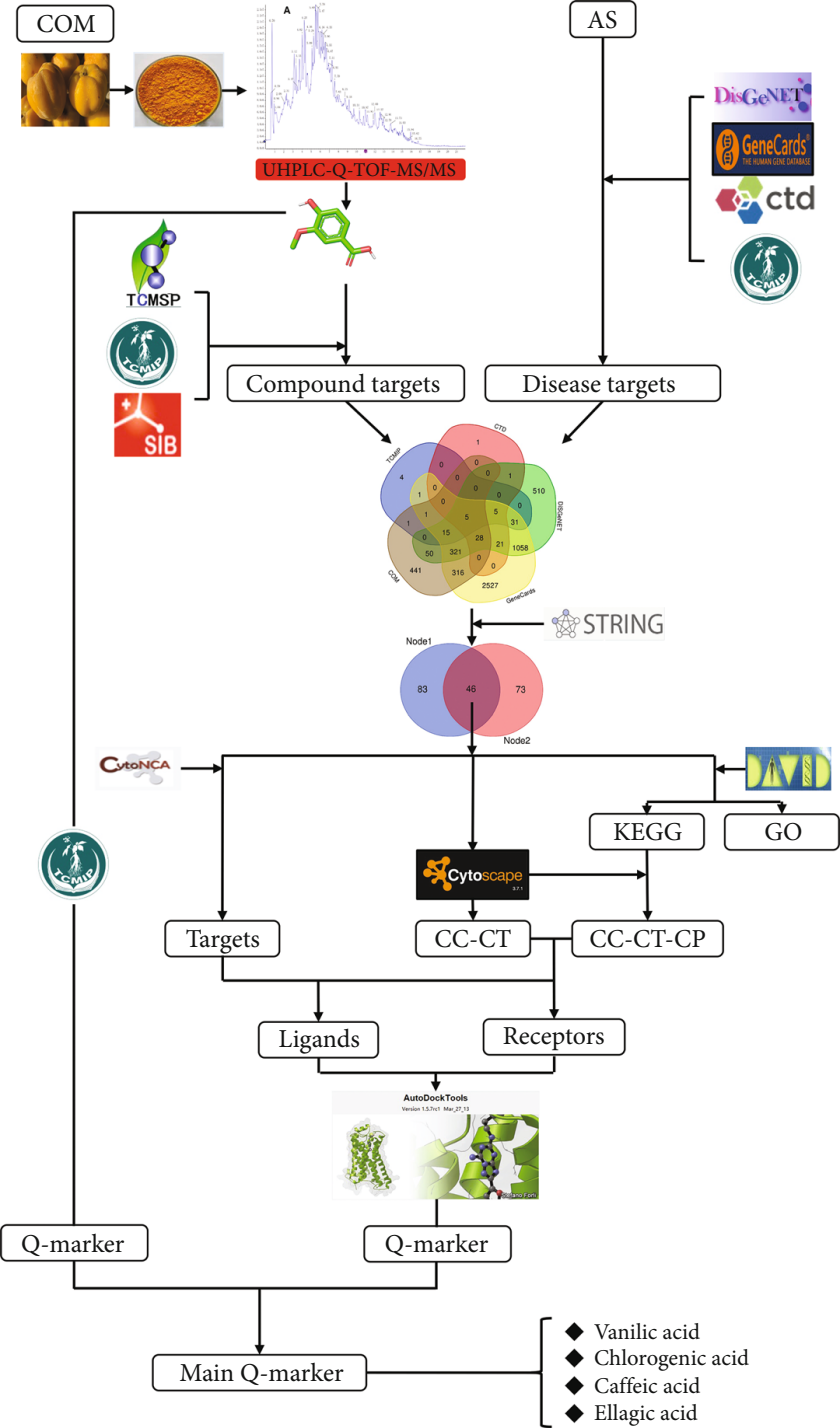
Network pharmacology workflow combined with UHPLC-Q-TOF-MS/MS.

The non-targeted metabolism of TCM components emphasizes the *in vivo* study, which is very similar to the principles of the holistic and dynamic nature of TCM. UHPLC Q-TOF-MS/MS has been widely used for the multicomponent analysis of drugs for its sharp specificity, fast speed, and strong selectivity [11]. Therefore, in this study, UHPLC Q-TOF-MS/MS is applied to identify the chemical composition of the extract, and then, potential Q-markers of COM are predicted for treating AS, based on the designed specific quality control methods mentioned above. The design of this experiment is shown in Figure 1.

## 2. Materials and Methods

### 2.1. Plant Material, Chemicals, and Reagents

COM was purchased from Yecheng County, Kashgar, Xinjiang Uyghur Autonomous Region, and was identified by Professor Parida Abuliz. The voucher specimens were stored at the Center Laboratory of Xinjiang Medical University, China. Chromatographic grade acetonitrile, methanol, and formic acid were purchased from Merck (Darmstadt, Germany). The Rutin standard sample is purchased from Shanghai Yuanye Biotechnology Co., Ltd. 95% ethanol is purchased from Tianjin Fengchuan Chemical Reagent Technology Co., Ltd. AB-8 macroporous adsorption resin is purchased from Cangzhou Baoen Adsorption Materials Technology Co., Ltd. The ultraviolet-visible spectrophotometer is purchased from Shimadzu, Japan; Milli-Q Integral from Millipore, USA; UHPLC (Nexera UHPLC LC-30A; Shimadzu); high-resolution mass spectrometry (Triple TOF 5600; AB Sciex); centrifuge (Heraeus Fresco17; Thermo Fisher Scientific); analytical balance (BSA124S-CW; Sartorius); grinder (JXFSTPRP-24; Shanghai Jingxin Technology Co., Ltd.); ultrasonic apparatus (PS-60AL; Shenzhen Redbang Electronics Co., Ltd.); chromatographic column (ACQUITY UPLC BEH C18 1.7 *μ*m 2.1∗100 mm; Waters).

### 2.2. Preparation of Total Flavonoids from COM

The COM extraction process referenced previous methods mentioned: the carefully washed COM fruit was sliced into pieces, dried in the shade, and crushed through 20-mesh sieves to gather the powder. Powder was soaked with 10 times the amount of 60% ethanol for 4 hours, then using ultrasonic extraction 2 times 40 min each time, combined with filtrate, vacuum concentration to extract. A certain amount of extract was dissolved in water and filtered, and AB-8 macroporous adsorption resin was used on the filtrate. After full adsorption, the water-soluble polar components of protein carbohydrates were eluted with distilled water and then eluted with 50% ethanol and 90% ethanol. The liquid was combined and concentrated, and the total flavonoid powder of COM fruit was obtained after freeze-drying. The content of total flavonoids in COM extract was determined by the UV spectrophotometer with Rutin as the standard substance, with a content of 38.61%. The powder was placed in a dryer and was stored in a cool place for further use.

### 2.3. UHPLC-Q-TOF-MS/MS Analysis of Chemical Constituents from COM Extract

#### 2.3.1. Preparation of Test Solution

Take a certain amount of COM extract with pure water prepared in constant volume, COM extract solution (4 mg/ml); take a certain amount of solution and centrifuge in 13000 rpm for 10 min; take out supernatant and filter with a 0.22 *μ*m microporous membrane for injection.

#### 2.3.2. Chromatographic Condition

The column was Waters UHPLC BEH C18 (1.7 *μ*m∗2.1∗100 mm). The mobile phase was 0.1% formic acid water (A)-acetonitrile (B) with gradient elution (0-3.5 min, 5%-15% B; 3.5-6.0 min, 15%-30% B; 6.0-6.5 min, 30% B; 6.5-12 min, 30%-70% B; 12-12.5 min, 70% B; 12.5-18 min, 70%-100% B; and 18–22 min, 100% B), flow rate of 0.4 *μ*l/min, column temperature of 40°C, injection volume of 5 *μ*l, and full wavelength scanning of 200-600 nm.

#### 2.3.3. Mass Spectrometer Conditions

AB 5600 Triple TOF mass spectrometer is based on IDA function for primary and secondary mass spectrometry data acquisition. Bombardment energy is 40 eV, collision energy difference is 20 V, and there are 15 secondary spectra per 50 ms. ESI ion source parameters are as follows: atomization pressure (GS1): 55 Psi, auxiliary pressure: 55 Psi, curtain pressure: 35 Psi, temperature: 550°C, and spray voltage: 5500 V (positive ion mode) or -4000 V (negative ion mode). In each data acquisition cycle, the strongest and more than 100 molecular ions were selected to collect the corresponding secondary mass spectrometry data. The original mass spectrometry was imported by Progenesis QI software. The retention time correction, peak recognition, extraction, and integration were carried out by MAPS software. The peaks of MS/MS data were identified by the Biotree self-built secondary mass spectrometry database and the corresponding pyrolysis law matching method.

### 2.4. Data Analysis

SPSS 21.0 statistical software was used for data processing. Data are expressed as the mean ± standard deviation. Analysis of variance was used to compare the orthogonal experiment between groups; *P* < 0.05 was considered statistically significant.

### 2.5. Preliminary Screening of COM Intervention on AS Q-Markers

#### 2.5.1. Screening of COM Targets

The COM chemical components obtained by UHPLC-Q-TOF-MS/MS analysis were input into the database of traditional Chinese medicine components of Integrative Pharmacology-based TCMIP V2.0 (http://www.tcmip.cn/TCMIP/index.php). The above COM components were used as the limiting words. According to the molecular fingerprint feature extraction method of MACCS (molecular ACCess system) and the similarity measurement method defined by the Tanimoto coefficient, the similarity scores of the chemical components of traditional Chinese medicine and the drugs listed in FDA were scored. When the score was ≥0.8, the desired targets were extracted to avoid the screening of components with high recognition; the targets of COM components were searched by TCMSP (http://lsp.nwu.edu.cn/tcmsp.php) and SwissTargetPrediction (http://www.swisstargetprediction.ch/). Due to the problems of irregular naming of drug targets retrieved from the three databases, this paper uses the UniProtKB search function in the protein synthesis database (UniProt, https://www.uniprot.org/) to correct all the retrieved targets to their official names by inputting the name of the target and limiting the species to “human.”

#### 2.5.2. Collection of AS Targets

The term “atherosclerosis” was used in the TCMIP V2.0 disease-related molecular library. The DisGeNET database (http://www.disgenet.org) (with “UMLS CUI: C0004153” as the limiting word) and GeneCards database (http://www.genecards.org/) were searched, and the Comparative Toxicogenomics Database (CTD) (http://ctdbase.org/) (with “marker/mechanism and therapeutic” as the limiting word) was combined to retrieve and screen known AS disease targets and delete repeated targets, to obtain known targets in the pathogenesis of AS. The AS disease target was mapped to the action target of COM active components to obtain the action target of COM against AS.

#### 2.5.3. Construction of Protein Interaction Network (PPI)

In String (http://genemania.org/), species selection “Homo sapiens,” and ^“^minimum required interaction score^”^ ≥ 0.999, the rest were used to analyze the interaction between each target by default parameters. In the construction of protein-protein interaction (PPI), delete isolated nodes to get the initial network. The intersection of “Node1” and “Node2” was obtained, and the intersection target was a target with strong interaction among proteins, which was used as a candidate target for COM treatment of AS. The candidate targets of COM anti-AS and the COM components acting on this candidate were used to construct a visualized association integration network of “Candidate Component-C Candidate Target, CC-CT” by Cytoscape software. The common target network of COM-AS was calculated by the Cytoscape plug-in Network Analyzer, and the target was the main target of COM in the treatment of AS. The possible quality markers of COM intervention AS in Xinjiang COM were analyzed and excavated, and the preliminary screening of quality markers of COM intervention AS in Xinjiang was completed.

### 2.6. Gene Ontology (GO) and Kyoto Encyclopedia of Genes and Genomes (KEGG) Enrichment Analysis

“Effectiveness” is the core element of quality marker determination. Because the control of the effectiveness of TCM is the fundamental purpose of quality control, the discovery of disease-related chemical components of traditional Chinese medicine as candidate quality markers from the network diagram of “traditional Chinese medicine-composition-target-pathway” by network pharmacology is the key idea for effective efficacy research. Gene function (GO) and pathway enrichment analysis of COM anti-AS candidate targets were performed using DAVID (version 6.8, https://david.ncifcrf.gov/) with the error detection rate (FDR) as screening criteria. GO gene functions include biological process (BP), molecular function (MF), and cellular component (CC) analysis. The smaller the FDR value, the higher the enrichment of GO and KEGG. Ranked by FDR values, the top-down pathway is the key pathway. Finally, the “Candidate Compound-Candidate Target-Candidate Pathway, CC-CT-CP” network diagram was constructed by Cytoscape software, and the targets and their active components involved in the key signaling pathways were visually analyzed by degree to screen the main components.

### 2.7. Determination of Quality Markers Based on Component Transfer and Traceability

In this study, the TCMIP V2.0 database was used to sort out the physical and chemical properties of the chemical components that might be preliminarily screened as quality markers, including absorption level and solubility, and further confirm the quality markers of Xinjiang COM intervention in AS combined with its common clinical medication and extraction process.

### 2.8. Determination of Quality Markers Based on Molecular Docking

In the determination process of Q-markers, the chemical composition “measurability” is the necessary prerequisite. The candidate targets used the CytoNCA (2.1.6) plug-in to simplify the network with the median ≥ 2 of topological properties such as betweenness centrality (BC), closeness centrality (CC), eigenvector centrality (EC), degree centrality (DC), local average connectivity-based method (LAC), and network centrality (NC) of initial network nodes as screening criteria, and the obtained targets were used as potential targets for COM in the treatment of AS.

The preliminary screening components of the integrated pharmacology platform were sorted out through molecular docking technology. The main targets obtained under “2.5.3” and “2.6” and the potential targets obtained by the above network topology parameters such as BC, CC, EC, DC, LAC, and NC are mapped to the intersection core targets as receptors. In the “CC-CT” and “CC-CT-CP” networks obtained under “2.5.3” and “2.6,” respectively, the components screened by degree were intersected, and the main components of the intersection were used as ligands for molecular docking. Receptors and ligands are docked via Autodock Vina (version 1.2, http://vina.scripps.edu/index.html). COM components were confirmed by PubChem (https://www.ncbi.nlm.nih.gov) and Chemical Book database (https://www.chemicalbook.com/). Files in mol format are saved through the Chemical Book database. The structural components were loaded into the AutoDock Tools 1.5.6 program, and atomic charges were added to distribute atomic types. All flexible bonds were rotated by default as docking ligands. AutoTools software was used to pretreat the protein crystal structure of the core targets, remove the redundant protein chains and ligands, remove water molecules by hydrogenation, and calculate the Gasteiger charge as the receptor for molecular docking. Then, Autodock Vina software was used for docking small molecules with proteins. Finally, we complete the conformation analysis and mapping.

Binding energy (BE) is used to determine the matching degree between COM components and core targets. When the ligand and receptor conformation is stable, the lower the energy, the greater the possibility of interaction. In general, BE ≤ −4.25 kcal/mol indicates that the active ingredient has certain binding energy with the target, BE ≤ −5.00 kcal/mol indicates that the active ingredient has stable binding energy with the target, and BE ≤ −7.00 kcal/mol indicates that the active ingredient has strong binding energy. In this paper, BE ≤ −5.00 kcal/mol was selected as the standard, and the components with good docking with the main target of COM in the treatment of AS were finally obtained as the Q-marker of COM in the treatment of AS. Finally, the Q-marker was crossed with the Q-marker obtained under “2.5.3” and “2.6,” and the common Q-marker was used as the main quality marker for COM intervention in AS.

## 3. Results

### 3.1. Analysis of Chemical Components of COM Extract

**Figure 2:**
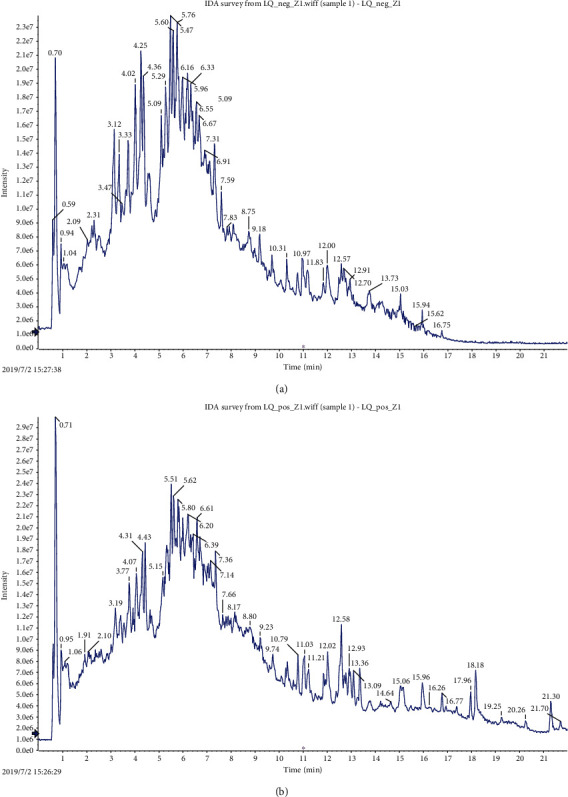
Total ion chromatograms of COM extracts: (a) negative ion mode (-) ESI; (b) positive ion mode (+) ESI.

Total ion chromatogram (TIC) refers to the figure of the total time or scanning times of all ions after the intensity of all ions which is added within the selected mass range. All the compounds detected by UHPLC-Q-TOF-MS/MS were retrieved from the Biotree DB database and compared with the references. The chemical components observed in the extract were analyzed by integrating the first-order and second-order mass spectra under (+) ESI and (−) ESI modes. A total of 72 compounds were identified, including 39 compounds in negative ion mode and 33 compounds in positive ion mode. The total ion flow diagram of positive and negative ions and the list of identification components are shown in Figures 2(a) and 2(b) and Table 1.

### 3.2. Preliminary Screening of COM Intervention on AS Quality Markers

**Figure 3:**
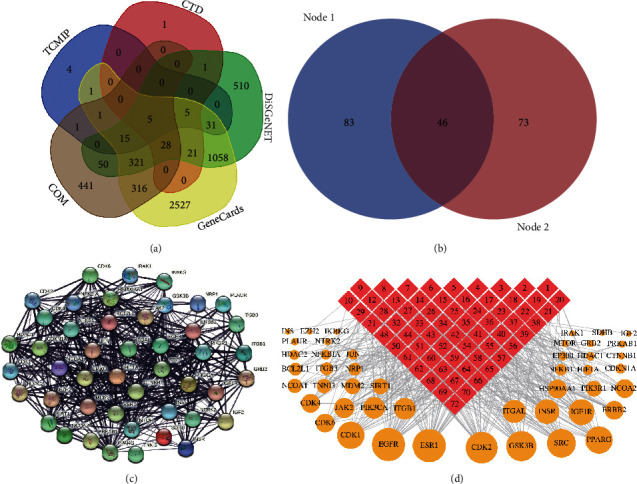
Preliminary screening of AS quality markers for COM intervention. (a) Venn diagram crossover: 737 targets are the common points of AS disease targets obtained from COM and TCMIP, CTD, DiSGeNET, and GeneCards databases; (b) combination of Venn diagram: 46 candidate targets were screened with the minimum required interaction score ≥ 0.999; (c) PPI network diagram: including 46 nodes and 518 edges; (d) component-target network, including 110 nodes and 437 edges, the yellow circle represents 46 candidate targets, the red square represents 64 COM components, and the size of the circle represents the node degree of the target protein.

The above 72 components collected by TCMIP, TCMSP, and SwissTargetPrediction database were obtained, respectively, 243, 133, and 1017 targets. After deleting repetitive targets, 1202 targets were obtained. The TCMIP, CTD, DiSGeNET, and GeneCards databases were used to search for 65, 62, 2044, and 4329 disease targets, respectively, related to the pathogenesis of AS, and 737 common targets were obtained by crossing with 1202 COM targets, as shown in Figure 3(a). A total of 737 drug-disease common targets were screened by String data using “Homo sapiens” and ^“^minimum required interaction score^”^ ≥ 0.999 as criteria, and 46 candidate targets were screened. The interaction between the targets is shown in Figure 3(b), and there is a correlation between the two targets. The network contains 46 nodes and 518 edges (Figure 3(c)). 46 candidate targets were connected to COM components, and the component-target network diagram was constructed by Cytoscape software. The network consists of 110 nodes (including 64 components and 46 candidate targets) and 437 edges. The edges between components (red quadrangle) and candidate targets (yellow circle) represent interactions (Figure 3(d)).

Nodes represent proteins, and edges represent the correlation of functions. The more lines represent the greater correlation. In addition, the degree of COM-AS common target network was calculated by the Cytoscape plug-in network analyzer with a degree as the screening condition. The top 10 targets with higher degrees include estrogen receptor (ESR1, degree = 36), epidermal growth factor receptor (EGFR, degree = 36), cell division protein kinase 2 (CDK2, degree = 32), glycogen synthase kinase-3 beta (GSK3B, degree = 26), protooncogene tyrosine-protein kinase SRC (SRC, degree = 24), peroxisome proliferator-activated receptor gamma (PPARG, degree = 2), cell division control protein 2 homolog (CDK1, degree = 20), insulin-like growth factor 1 receptor (IGF1R, degree = 19), insulin receptor (INSR, degree = 18), and integrin alpha-L (ITGAL, degree = 18). It can be the main target of COM for the treatment of AS.

The initial screening of the 64 components of COM was performed according to the screening criterion of degree ≥ 6, and the obtained components were used as the initial screening results for the COM intervention AS quality markers. As shown in Table 2, 46 components, such as ellagic acid (degree = 18), pectolinarigenin (degree = 14), eupatilin (degree = 14), acacetin (degree = 13), and ursolic acid (degree = 12), were obtained as the outcome of the initial screening of COM intervention AS quality markers.

### 3.3. GO and KEGG Enrichment Analysis Results

**Figure 4:**
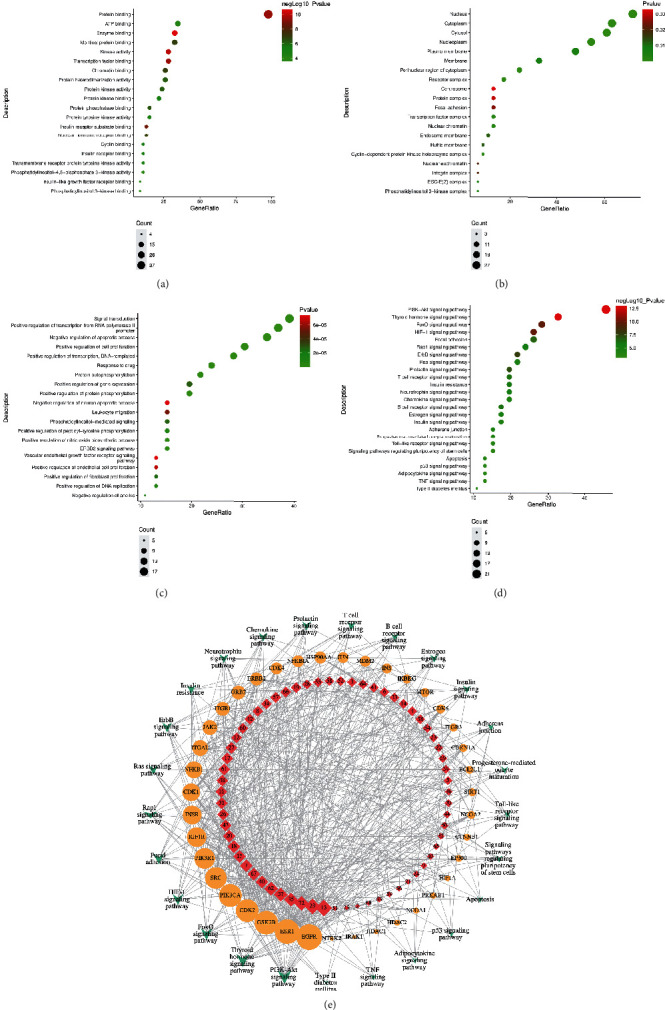
COM intervention in the determination of AS quality markers. (a) The first 20 molecular functions; (b) composition of the first 20 cells; (c) the first 20 biological processes; (d) the first 25 signaling pathways; (e) CC-CT-CP network diagram, red quadrangle represents 63 components; yellow circle represents 39 candidate targets; green diamond represents 25 signaling pathways; the greater the circle size, the greater the degree of representative components, targets, and pathways, the more important the network.

The results of GO gene function analysis of 46 candidate targets by DAVID showed that a total of 426 items were obtained, including 301 BP, 80 MF, and 45 CC, and the top 20 results were sorted according to the corrected FDR pairs as shown in Figure 4.

The top 10 biological functions induced by the 64 COM components include enzyme binding, kinase activity, protein binding, transcription factor binding, insulin receptor substrate binding, nuclear hormone receptor binding, chromatin binding, identical protein binding, protein phosphatase binding, and protein heterodimerization activity (Figure 4(c)). The top 10 cell components listed include cytosol, nucleoplasm, nucleus, receptor complex, perinuclear region of cytoplasm, cytoplasm, cyclin-dependent protein kinase holoenzyme complex, nuclear chromatin, transcription factor complex, and plasma membrane (Figure 4(b)). The top 10 biological processes include negative regulation of apoptotic process, positive regulation of cell proliferation, protein autophosphorylation, ERBB2 signaling pathway, positive regulation of protein phosphorylation, positive regulation of nitric oxide biosynthetic process, positive regulation of transcription from RNA polymerase II promoter, signal transduction, response to drug, positive regulation of transcription, and DNA-templated (Figure 4(a)).

Forty-six candidate targets were imported into the DAVID for analysis of KEGG signaling pathways, and the pathways were further screened according to FDR size, with the FDR value indicating that the smaller the value in the enrichment analysis, the higher the enrichment significance [12]. A total of 83 signaling pathways were collected and sorted according to FDR ≤ 0.05, and the top 25 results are shown in Figure 4(d), including the PI3K-Akt signaling pathway (hsa04151), thyroid hormone signaling pathway (hsa04919), HIF-1 signaling pathway (hsa04066), FoxO signaling pathway (hsa04068), ErbB signaling pathway (hsa04012), prolactin signaling pathway (hsa04917), focal adhesion (hsa04510), T cell receptor signaling pathway (hsa04660), B cell receptor signaling pathway (hsa04662), insulin resistance (hsa04931), Rap1 signaling pathway (hsa04015), neurotrophin signaling pathway (hsa04722), estrogen signaling pathway (hsa04915), adherens junction (hsa04520), Ras signaling pathway (hsa04014), progesterone-mediated oocyte maturation (hsa04914), chemokine signaling pathway (hsa04062), insulin signaling pathway (hsa04910), apoptosis (hsa04210), Toll-like receptor signaling pathway (hsa04620), p53 signaling pathway (hsa04115), adipocytokine signaling pathway (hsa04920), type II diabetes mellitus (hsa04930), signaling pathways regulating pluripotency of stem cells (hsa04550), and TNF signaling pathway (hsa04668). Among them, the PI3K-Akt signaling pathway and the thyroid hormone signaling pathway presented a small FDR, representing a higher degree of significant enrichment.

To further determine the quality markers identified for COM treatment of AS, a component-target-pathway network map was constructed by Cytoscape software, which contains 127 nodes (including 63 candidate components, 39 candidate targets, and 25 signaling pathways) and 618 edges. The edges between components (red quadrangles), candidate targets (yellow circles), and signaling pathways (green diamonds) represent interactions. The larger the size of the circles, the larger the degrees of the components, targets, and pathways represented, the more important is the network.

The top 10 targets with high degrees include EGFR (degree = 45), ESR1 (degree = 39), GSK3B (degree = 37), phosphatidylinositol 4,5-bisphosphate 3-kinase catalytic subunit alpha isoform, PI3-kinase subunit alpha, PI3K-alpha, PI3Kalpha, PtdIns-3-kinase subunit alpha (PIK3CA, degree = 34), CDK2 (degree = 34), SRC (degree = 32), phosphatidylinositol 3-kinase regulatory subunit alpha (PIK3R1, degree = 30), IGF1R (degree = 28), INSR (degree = 27), and cell division control protein 2 homolog (CDK1, degree = 21). It can be a candidate target for COM treatment of AS.

The 63 components of COM were screened according to the screening criterion of degree ≥ 5, and the obtained components were used as rescreening results for the COM intervention AS quality markers. As shown in Table 3, 49 components were obtained for ellagic acid (degree = 16), acacetin (degree = 12), ursolic acid (degree = 11), acacetin (degree = 10), and eupatilin (degree = 10).

### 3.4. Quality Marker Results Based on Quality Transfer and Traceability

The common physicochemical properties of the above components such as oil-water partition coefficients (data sources are available in the TCMIP V2.0 chemical composition database) were summarized in conjunction with the TCMIP V2.0 chemical composition database, and the specific parameters are shown in Table 4. Molecular solubility and ADMET absorption level of the compounds were calculated by the TCMIP using Pipeline Pilot software (version 7.5), representing the molecular solubility and its absorption level *in vivo*, respectively. The optimal lg*P* value for a drug is −1 < lg*P* < 2 for better absorption [13]. In this paper, lg*P* values of −1 < lg*P* < 2 and ADMET absorption level > 0 were used as criteria for screening to obtain vanillic acid, chlorogenic acid, cryptochlorogenic acid, isolariciresinol-4-O-Î′-D-glucopyranoside, ellagic acid, luteolin-4′-O-glucoside, isochlorogenic acid B, sophoricoside, caffeic acid, and eucommin A, which can be used as quality markers for COM intervention in AS.

### 3.5. Molecular Docking-Based Quality Markers Redefined

**Figure 5:**
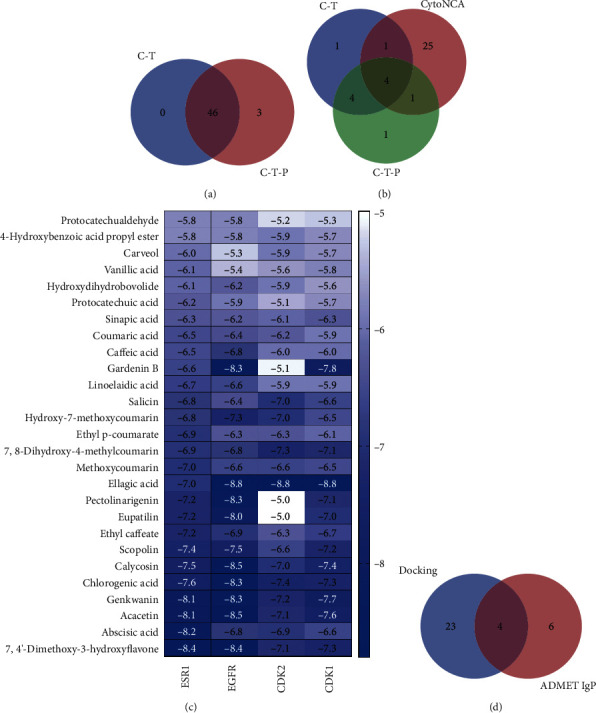
Rescreening of AS quality markers by COM intervention. (a) The intersection of ligands: 46 components are the common points of components obtained by “C-T” and “C-T-P” network analysis. (b) The intersection of receptors: 5 targets are the common points of candidate targets obtained by “C-T” and “C-T-P” network analysis. (c) Docking results (BE ≤ −5.00 kcal/mol): docking of 25 components with 4 core targets, the darker the color, the stronger the receptor and ligand binding activity. (d) The main Q-marker intersection map: the common points of 10 components screened by physicochemical properties and 27 core components with good docking results.

The 46 candidate targets were simplified by using the CytoNCA plug-in for the initial network nodes with the median ≥ 2 of the topological properties of BC, CC, EC, DC, LAC, NC, etc. The screening criteria and the 31 potential targets obtained as potential targets for COM treatment of AS are shown in the Table 5. The “CC-CT” and “CC-CT-CP” obtained under “3.2” and “3.3,” respectively, in the “CC-CT” and “CC-CT-CP” networks, and the top 10 candidate targets with higher degrees of each network were intersected with 31 potential targets, and the obtained intersected targets were used as receptors for molecular docking. As can be seen in Figure 5(b), the core targets of the intersection include EGFR (PDBID=5GTY), ESR1 (PDBID=4XI3), CDK1 (PDBID=6GU6), and CDK2 (PDBID=1B39).

In the “CC-CT” and “CC-CT-CP” networks obtained under “3.2” and “3.3,” respectively, network, the 46 candidate components obtained according to degree screening were intersected with 49 candidate components, which can be seen in Figure 5(a), and 46 core components were obtained, and the obtained core components were used as ligands for molecular docking with 4 core targets, as shown in Tables 6 and 7. In this paper, with BE ≤ −5.00 kcal/mol, 27 of the 46 core components had good binding activity to the 4 core targets, shown in Figure 5(c).

The 27 core components that dovetailed well with the 10 components obtained based on “3.4” were intersected to obtain 4 components as the main Q-markers for COM for AS, which are shown in Figure 5(d), including caffeic acid, chlorogenic acid, ellagic acid, and vanillic acid. The specific binding molecular models of the four main Q-markers to the four core targets, including EGFR, ESR1, CDK1, and CDK2.

**Figure 6:**
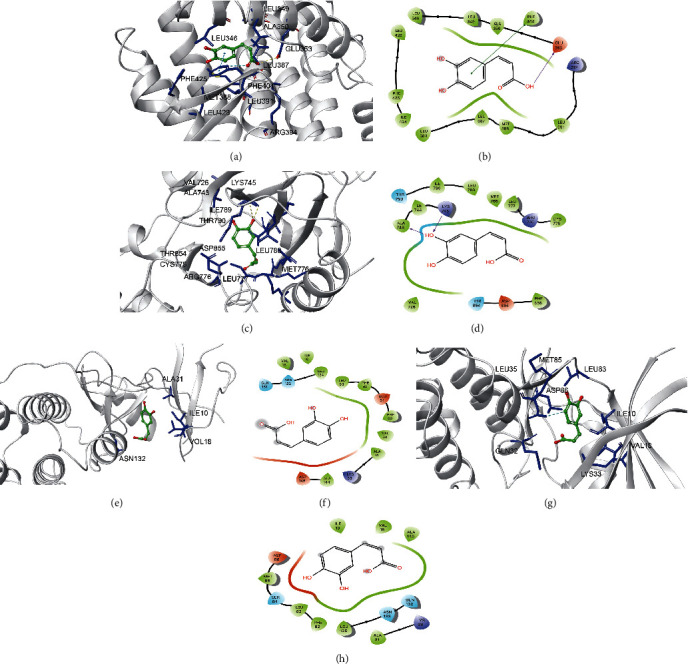
Molecular models of the binding of caffeic acid to the predicted targets ESR1 (a, b), EGFR (c, d), CDK2 (e, f), and CDK1 (g, h) shown as 3D diagrams and 2D diagrams.

Caffeic acid can form hydrophobic interactions with adjacent residues LEU349, ALA350, LEU346, LEU525, HIS524, GLY521, MET421, TRP383, LEU384, MET388, LEU391, ARG394, ILE424, and PHE425 by forming hydrophobic interactions with PHE404 and GLU353 (3.0 Å) to form hydrogen bonds and *π* interactions with PHE404 (3.0 Å) to bind to ESR1 (Figures 6(a) and 6(b)). Caffeic acid binds to EGFR (VAL726, ILE789, THR790, LEU788, THR854, CYS775, ASP855, ARG776, LEU777, and MET776) at the active site, and hydrogen bonds formed with LYS745 and ALA743 (3.0 Å) further enhanced the interaction between the ligand and EGFR protein (Figures 6(c) and 6(d)). Caffeic acid can form a hydrogen bond with the adjacent residues ASN132, ILE10, VAL18, and ALA31. Caffeic acid can bind to CDK2 by forming hydrophobic interactions with adjacent residues MET785, LEU83, LEU135, GLN132, ILE10, VAL18, and LYS33 and hydrogen bonds with ASP86 (3.0 Å) (Figures 6(g) and 6(h)).

**Figure 7:**
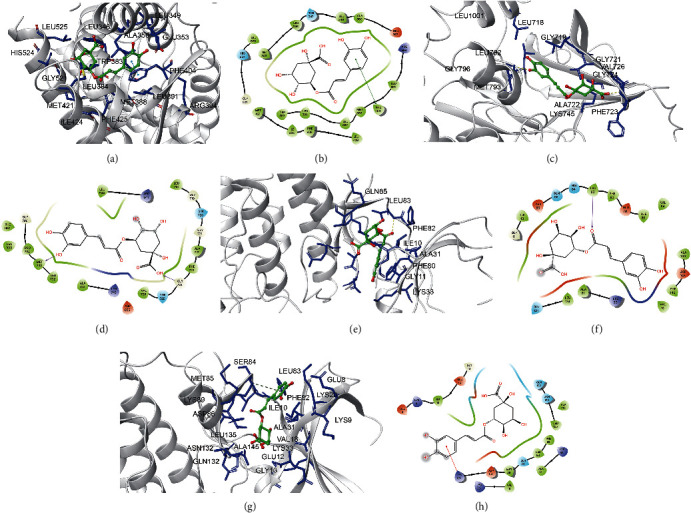
Molecular models of the binding of chlorogenic acid to the predicted targets ESR1 (a, b), EGFR (c, d), CDK2 (e, f), and CDK1 (g, h) shown as 3D diagrams and 2D diagrams.

Chlorogenic acid can bind to ESR1 by forming a *π* interaction with adjacent residues LEU349, GLU353, ALA350, LEU346, LEU525, HIS524, GLY521, MET421, TRP383, LEU384, MET388, LEU391, ARG394, ILE424, and PHE425. Caffeic acid binds to ESR1 by forming hydrophobic interactions with PHE404 (3.0 Å) (Figures 7(a) and 7(b)). Caffeic acid binds to the active sites of EGFR (GLY796, LEU1001, LEU718, GLY719, PHE723, LEU792, GLY721, VAL726, ALA722, and LYS745) and hydrogen bonds formed with MET793 and GLY724 (3.0 Å), further enhancing the interaction between the ligands and EGFR proteins (Figures 7(c) and 7(d)). Caffeic acid bound to CDK2 (GLN65, ASP86, LEU134, GLN131, VAL64, ALA144, ASP145, LYS33, PHE82, ILE10, ALA31, PHE80, GLY11, and VAL18) at the active site and hydrogen bonds formed with LEU83 (3.0 Å), further enhancing the interaction between the ligand and CDK2 protein (Figures 7(e) and 7(f)). Chlorogenic acid can be activated by binding to adjacent residues SER84, MET85, LEU83, ASP86, GLU12, LYS33, LEU135, PHE82, GLN132, ASN132, GLY13, ALA145, GLU8, LYS20, ILE10, ALA31, LYS9, and VAL18 and forming hydrophobic interactions with LYS89 (4.0 Å) to form *π* interactions, thus binding to CDK1 (Figures 7(g) and 7(h)).

**Figure 8:**
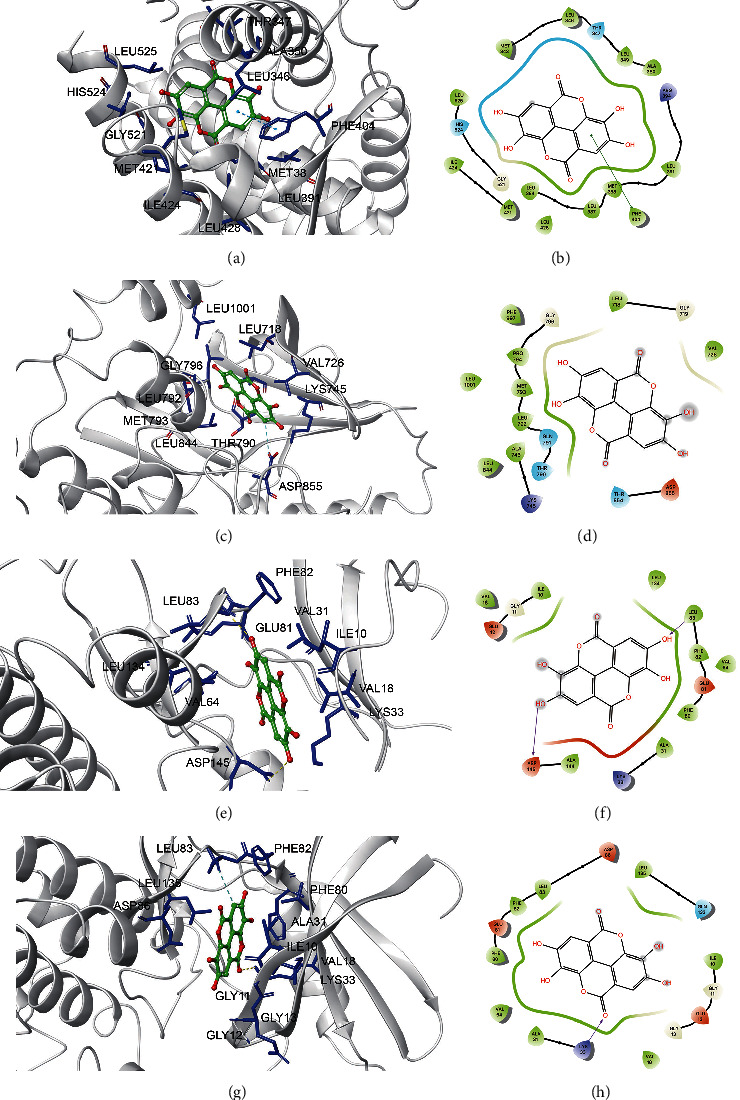
Molecular models of the binding of ellagic acid to the predicted targets ESR1 (a, b), EGFR (c, d), CDK2 (e, f), and CDK1 (g, h) shown as 3D diagrams and 2D diagrams.

Ellagic acid can form hydrophobic interactions with adjacent residues LEU525, HIS524, GLY521, MET421, ILE424, MET388, LEU391, LEU346, ALA350, THR347, and LEU428 and *π* interactions with PHE404 (3.0 Å), resulting in ESR1 binding (Figures 8(a) and 8(b)). Ellagic acid binding at the active site of EGFR (LEU1001, LEU718, VAL726, LYS745, ALA743, THR790, GLY796, LEU792, MET793, and LEU844) and hydrogen bonding with ASP855 (3.0 Å) further enhanced the interaction between the ligand and EGFR proteins (Figures 8(c) and 8(d)). Ellagic acid binding at the active site of CDK2 (PHE82, GLN81, LEU134, VAL64, LYS33, VAL18, VAL31, and ILE10) and hydrogen bonding with ASP145 and LEU83 (3.0 Å) further enhance the interaction between the ligand and CDK2 protein (Figures 8(e) and 8(f)). Ellagic acid can interact with CDK2 by forming hydrophobic interactions with adjacent residues PHE82, LEU135, ASP86, PHE80, ALA31, ILE10, VAL18, GLY11, GLY13, and GLU12 and with LYS33 and LEU83 (3.0 Å) to form hydrogen bonds, thus binding to CDK1 (Figures 8(g) and 8(h)).

**Figure 9:**
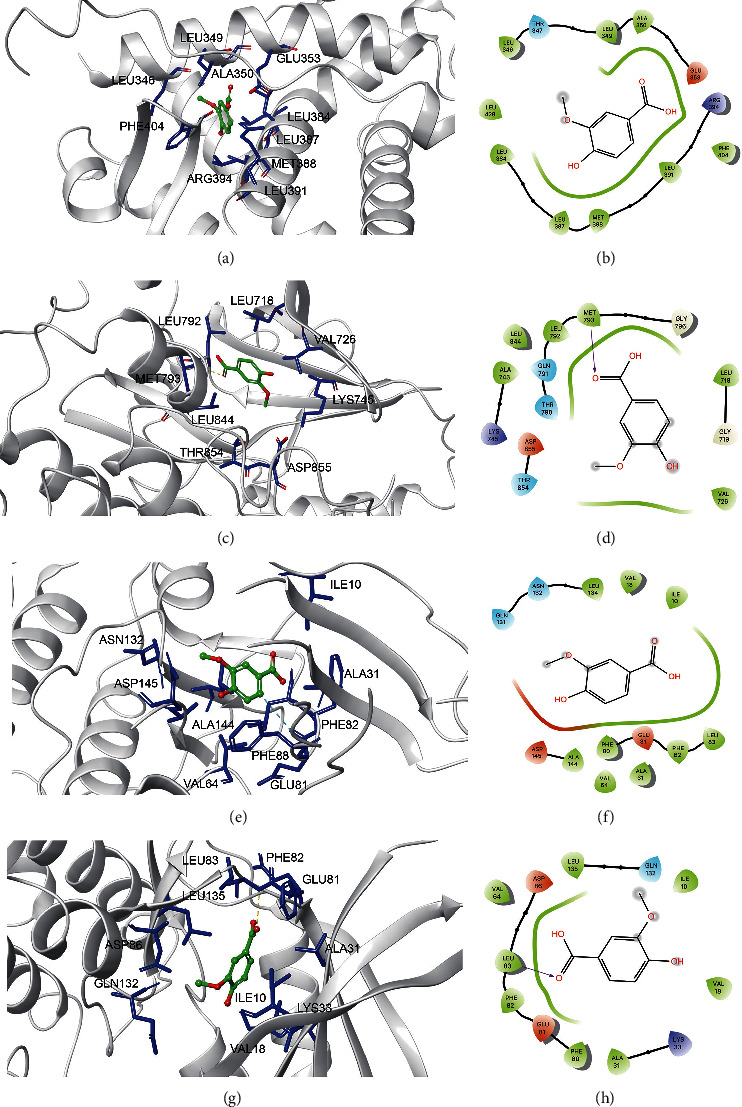
Molecular models of the binding of vanillic acid to the predicted targets ESR1 (a, b), EGFR (c, d), CDK2 (e, f), and CDK1 (g, h) shown as 3D diagrams and 2D diagrams.

Vanillic acid can bind to ESR1 by forming hydrophobic interactions with adjacent residues LEU349, ALA350, GLU353, LEU346, PHE404, LEU384, LEU387, MET388, LEU391, and ARG394 (Figures 9(a) and 9(b)). Vanillic acid binds to the active site of EGFR (LEU718, VAL726, LYS745, ASP855, THR854, LEU844, and LEU792) and hydrogen bonds formed with MET793 (3.0 Å), further enhancing the interaction between the ligand and EGFR protein (Figures 9(c) and 9(d)). Vanillic acid binds to CDK2 (ASN132, ASP145, ALA144, LEU134, VAL64, PHE88, PHE82, LEU83, ALA31, and ILE10) at the active site, and hydrogen bonds formed with GLU81 (3.0 Å) further enhanced the interaction between the ligand and CDK2 protein (Figures 9(e) and 9(f)). Vanillic acid can bind to CDK1 by forming hydrophobic interactions with adjacent residues PHE82, GLU81, LEU135, ASP86, GLN132, ILE10, VAL18, LYS33, and ALA31 and hydrogen bonds with LEU83 (4.0 Å) (Figures 9(g) and 9(h)).

## 4. Discussion

AS is a chronic progressive lesion of the aorta caused by disorders of lipid metabolism and inflammatory response, which is the pathological basis of several cardiovascular diseases. The previous research of our research group mainly focused on the pharmacodynamic effects of COM on cardiovascular diseases; however, the material basis and active components of AS prevention and treatment are not studied in depth. The nontarget metabolomics of TCM plays a leading effect on the modernization research of TCM, such as the discovery of TCM components, the study of the mechanism of action of prescriptions, and the discovery of drug targets, and the elucidation of metabolic pathways and mechanisms of action of TCM components are an important way for TCM to face and develop with the world. Currently, the development of UHPLC-Q-TOF-MS/MS analysis technology provides technical support for the study of mixed components of TCM [14, 15]. In this study, 72 components of COM and their 1202 action targets were obtained by UHPLC-Q-TOF-MS/MS technique, and this drug target intersected with AS disease targets, and 737 common drug-disease targets were obtained, and 46 candidate targets of COM against AS were obtained by PPI analysis. 46 candidate targets were screened by network topology parameters to obtain 31 potential targets. 46 candidate targets were enriched by network topology parameters to obtain 46 potential targets. The enrichment analysis of the 46 candidate targets yielded 426 GOs and 83 signaling pathways. The common candidate components obtained from “CC-CT” and “CC-CT-CP” were used as ligands, and 31 potential targets were used as receptors for docking, and the criteria of BE ≤ −5.00 kcal/mol were used for the 46 core components. The 27 active ingredients were intersected with 10 active ingredients obtained by mass transfer and traceability of quality markers to obtain four Q-markers of COM anti-AS, consisting of caffeic acid, chlorogenic acid, ellagic acid, and vanillic acid, all of which belong to phenolic acids. Phenolic acid components have strong anti-inflammatory, antioxidant, anti-free radical, and hypolipidemic effects [16, 17]. The present study predicts an anti-AS effect mainly by acting on EGFR, ESR1, CDK1, and CDK2.

Caffeic acid (CA) is a natural phenolic compound belonging to the family of polyphenolic phenolic acids with a hydroxybenzoic acid structure that is most abundant in fruits, vegetables, and beverages and has biological activities such as antioxidant, anti-inflammatory, antibacterial, and cytostatic [18]. CA modulates the renin-angiotensin-aldosterone endocrine axis in vitro [19], and in cyclosporine-induced hypertension experiments in Sprague-Dawley rats, CA significantly reduced plasma angiotensin-converting enzyme (ACE) activity [20]. Studies have shown the ability of CA to inhibit ACE activity and prevent the formation of advanced glycosylation end products (AGE) and its antioxidant, reducing, and chelating activities [21]. CA prevents the oxidation of LDL and the increase in calcium concentration, thus preventing apoptosis, and thus, CA may act as an inhibitor of LDL oxidation and may treat atherosclerosis [22]. However, the exact mechanism of action has not been clarified. The results of the present study suggest that CA can form stable hydrogen bonds and *π* interactions with amino acid residues of EGFR, ESR1, CDK1, and CDK2, the core targets associated with the pathogenesis of AS.

Activation of the epidermal growth factor receptor (EGFR) is closely linked to the physiology and pathophysiology of the cardiovascular system, and inhibition of EGFR activity is emerging as a potential therapeutic strategy for the treatment of diseases such as hypertension, cardiac hypertrophy, renal fibrosis, and abdominal aortic aneurysms [23]. Studies have shown that enhanced expression and plasma secretion of heparin-binding epidermal growth factor (HB-EGF), a ligand of EGFR, and enhanced arterial EGFR activation were observed in animal models of diet-induced atherosclerosis. Stanic et al. demonstrated for the first time that a causal relationship between EGFR activation and NADPH oxidase expression was studied and identified EGF-like ligands as a potential modulator of atherosclerosis [24]. In contrast, CA, the active component of COM anti-AS, can inhibit EGF-induced EGFR autophosphorylation, as well as the ability to inhibit oxidized low-density lipoprotein- (OxLDL-) induced cellular oxidative stress and participate in the activation of epidermal growth factor receptors [25]. The accuracy of the predicted results in this paper was further determined.

Estrogen receptor alpha (ESR1) is an important gene transcriptional regulator known to mediate the effects of estrogen. Alterations in ESR1 expression and its function affect the atheroprotective effects of circulating estrogen in insulinogenic atherosclerosis [26]. ESR1 plays an important role in the regulation of adipose tissue VEGFA, which is known to enhance vascular generation, reduce inflammation, and improve adipose tissue function which is potentially important [27]. In contrast, whether CA regulates the expression of ESR1 and thus the anti-AS effect has not been reported in the study, which is the next step to be validated by our group.

Cycle protein-dependent kinase 1 (CDK1) is one of the important kinases that drive cell entry into mitosis and is involved in cell cycle M phase, G2/M transition point, and G1 phase activities, as well as being a key protein for cell cycle passage through G1/S and G2/M phase restriction points. CDK1 inhibits microtubule-associated protein hydrolysis and regulates centrosome segregation and also promotes spindle assembly and centrosome maturation. Its overactivation, abnormal regulation of mitotic entry and progression, and deranged centrosome segregation accelerate cell proliferation [28]. CDK2 is a cell cycle protein-dependent kinase that remains at relatively constant levels throughout the cell cycle. Inhibition of CDK2 expression can significantly inhibit cell growth, causing cells to stay in the G1 phase while undergoing division, thus blocking cell proliferation. Blocking the vicious cycle of apoptosis and proliferation in AS can inhibit or even reverse the development of AS [29]. Caffeic acid phenethyl ester (CAPE) treatment, a natural derivative of CA, induces cell cycle arrest and growth inhibition in desmoplastic resistant prostate cancer (CRPC) cells by regulating Skp2, p53, p21Cip1, and p27Kip1 [30]. Among them, p27kip1 belongs to CDK1 which has a broad-spectrum inhibitory effect on CDK. SKP2 is a cell cycle G1/S transition and S-phase promotion through ubiquitination and degradation of CDK1. p53 and p21Cip1 are inhibitors of CDK2. In recent years, the mechanism of action of active ingredients of traditional Chinese medicine through blocking the cell cycle and thus inhibiting tumor growth has gradually become clear. It has been found that drugs that inhibit cell cycle progression have a therapeutic effect on AS [31]. Combining the above research reports with the present study, it can be speculated that whether CA, the active ingredient of COM, inhibits the cell cycle and thus exerts anti-AS effects needs to be further investigated.

Chlorogenic acid (CGA) is a phenolic compound composed of CA and quinic acid. CGA increased the mRNA levels of peroxisome proliferator-activated receptor gamma (PPAR*γ*), Liver X Receptor *α* (LXR*α*), ATP Binding Cassette Subfamily A Member 1 (ABCA1), and ATP Binding Cassette Subfamily G Member 1 (ABCG1) as well as the transcriptional activity of PPAR*γ*, resulting in an effective reduction of ApoE^−/−^ development of atherosclerosis in mice and promote cholesterol efflux from RAW264.7 macrophages [32, 33]. Lysophosphatidylcholine (LPC) is the main atherogenic compound that oxidizes LDL. CGA protects endothelial cells from LPC damage and thus inhibits atherosclerosis [34]. CGA inhibits MCF-7 cell proliferation by downregulating CyclinD1 expression and blocking cells in the G0/G1 phase. It can be speculated that whether CGA inhibits the cell cycle and thus exerts an anti-AS effect needs to be further investigated. However, studies on the effects of CGA on EGFR and ESR1 are less reported, and further studies are needed.

Ellagic acid (EA) a polyphenolic compound extracted from pomegranate fruit extract reduced the lipid accumulation and expression of key adipogenic genes peroxisome proliferator-activated receptor *γ* (PPAR*γ*), CCATT/enhancer-binding protein *α* (C/EBP*α*), sterol regulatory element-binding protein-1c (SREBP-1c), acetyl coenzyme A carboxylase (ACC), and fatty acid synthase (FAS) lipid accumulation and expression levels [35]. It also inhibits the gene expression of LPL mRNA and decreases the concentration of TC and TG in the blood and increases HDL, which may reduce the incidence of cardiovascular disease [36]. Wang et al. showed that EA inhibits the EGFR signaling pathway and thus the migration, invasion, and proliferation of melanoma cells [37]. However, whether EA exerts an anti-AS effect by inhibiting the EGFR signaling pathway is thus to be further investigated. It was shown that EA inhibits the activity of key transcription factors by inhibiting lipogenic inducers to induce the proliferation of pre- and post-3T3-L1 adipocytes, reducing the expression of cell cycle-related proteins [38]. It can be speculated as to whether CGA inhibits the CDK1 and CDK2 cell cycle and thus exerts anti-AS effects, which needs to be further investigated. However, studies on the role of CGA on ESR1 are less reported, and further studies are needed.

Vanillic acid (VA) is a phenolic compound in the oxidized form of vanillin, and VA has analgesic, antioxidant and anti-inflammatory, and neuroprotective effects on nuclear factor-*κ*B activation, proinflammatory cytokine production, oxidative stress, and acetylcholinesterase [39, 40]. Studies have shown that VA acts through the regulation of adhesion processes, E-selectin, and VEGF production, thus treating AS [41]. According to the results of this study, it can be speculated as to whether VA exerts its anti-AS effect through proteins such as EGFR, ESR1, CDK1, and CDK2, thus providing some research ideas for later studies.

## 5. Conclusions

In summary, this study targeted three core elements of traceability and deliverability, measurability, and efficacy, and applied the UHPLC-Q-TOF-MS/MS assay and network pharmacology methods to study the quality markers of COM, and finally identified that caffeic acid, chlorogenic acid, ellagic acid, and vanillic acid were identified as potential Q-markers for COM anti-AS, providing a reference for COM quality control. The method and strategy can also be extended and applied to compound preparation of other traditional Chinese medicines, as well as to provide new ideas for the quantitative evaluation and identification of quality markers.

## Figures and Tables

**Table 1:** Identification of the chemical constituents of the extracts from COM.

No.	Compound	Rt (min)	Ion mode	M/z value	MS/MS fragments	Molecular formula	Mass error (ppm)
1	Gluconic acid	0.67	neg	195.0511	195, 177, 129	C_6_H_12_O_7_	0.24
2	Malic acid	0.72	neg	133.0138	133, 115, 72	C_4_H_6_O_5_	-3.00
3	Catechol	2.02	neg	109.0297	109, 108, 91	C_6_H_6_O_2_	1.37
4	Vanillic acid	2.23	neg	167.0351	152, 108, 151	C_8_H_8_O_4_	0.44
5	Chlorogenic acid	2.30	neg	353.0865	191, 179, 135	C_16_H_18_O_9_	-3.74
6	Salicin	2.49	neg	331.1029	123, 121, 93	C_13_H_18_O_7_	-2.73
7	Protocatechualdehyde	2.77	neg	137.0241	137, 136, 119	C_7_H_6_O_3_	-2.41
8	Scopolin	3.30	neg	399.0922	191, 176, 161	C_16_H_18_O_9_	-3.20
9	Cryptochlorogenic acid	3.34	neg	353.0874	173, 179, 135	C_16_H_18_O_9_	-1.21
10	6,8-Di-C-glucopyranosylnaringenin	3.69	neg	595.1655	595, 385, 355	C_27_H_32_O_15_	-2.30
11	Lariciresinol-9-O-beta-D-glucoside	4.22	neg	521.2017	359, 344, 254	C_26_H_34_O_11_	-2.25
12	Coumaric acid	4.59	neg	163.0399	119, 93, 117	C_9_H_8_O_3_	-0.75
13	Ellagic acid	5.23	neg	300.9980	301, 283, 229	C_14_H_6_O_8_	-3.38
14	Isoswertisin 2″-O-beta-arabinoside	5.27	neg	577.1552	577, 284, 447	C_27_H_30_O_14_	-1.94
15	Rutin	5.41	neg	609.1457	609, 301, 271	C_27_H_30_O_16_	-0.72
16	Isovitexin 2″-O-rhamnoside	5.46	neg	577.1553	179, 221, 89	C_27_H_30_O_14_	-1.67
17	Isoorientin 6″-O-alpha-L-arabinoside	5.68	neg	579.1352	577, 285, 284	C_26_H_28_O_15_	-0.59
18	Luteolin-4′-O-glucoside	5.71	neg	447.0923	285, 284, 447	C_21_H_20_O_11_	-2.30
19	Kaempferol-3-O-rutinoside	5.73	neg	593.1514	593, 284, 285	C_27_H_30_O_15_	0.40
20	Alangilignoside D	5.77	neg	551.2131	551, 419, 404	C_27_H_36_O_12_	-0.53
21	Isochlorogenic acid B	6.10	neg	515.1194	173, 353, 179	C_25_H_24_O_12_	-0.12
22	Sophoricoside	6.28	neg	431.0969	268, 431, 269	C_21_H_20_O_10_	-3.43
23	Sapinofuranone A	6.33	neg	181.0868	57, 137, 119	C_10_H_14_O_3_	-1.24
24	Isolarisiresinol 2a-O-beta-D-glucopyranoside	6.37	neg	567.2082	341, 326, 61	C_26_H_34_O_11_	-0.21
25	Azelaic acid	6.46	neg	187.0972	125, 123, 187	C_9_H_16_O_4_	-1.78
26	Hydroxydihydrobovolide	6.55	neg	243.1230	73, 125, 243	C_11_H_18_O_3_	-4.16
27	Abscisic acid	6.90	neg	263.1286	204, 203, 219	C_15_H_20_O_4_	-0.95
28	Ethyl caffeate	7.89	neg	207.0665	133, 134, 135	C_11_H_12_O_4_	1.03
29	Acacetin	7.91	neg	283.0602	268, 283, 240	C_16_H_12_O_5_	-3.67
30	Calycosin-7-O-*β*-D-glucoside	7.94	neg	491.1187	283, 268	C_22_H_22_O_10_	-1.75
31	Pedunculoside	8.80	neg	695.4013	487, 695, 649	C_36_H_58_O_10_	0.21
32	Nordavanone	8.96	neg	227.1285	183, 165, 227	C_11_H_18_O_2_	-2.26
33	Ethyl p-coumarate	9.00	neg	191.0711	117, 119, 145	C_11_H_12_O_3_	-1.65
34	4-Hydroxybenzoic acid propyl ester	9.25	neg	179.0722	92, 93, 179	C_10_H_12_O_3_	4.46
35	Pectolinarigenin	9.46	neg	313.0715	283, 298, 313	C_17_H_14_O_6_	-0.82
36	Teuhetenone A	9.48	neg	239.1293	195, 239, 177	C_12_H_18_O_2_	1.98
37	Eupatilin	9.99	neg	343.0813	313, 298, 328	C_18_H_16_O_7_	-2.93
38	Calycosin	10.08	neg	283.0614	268, 283, 211	C_16_H_12_O_5_	0.80
39	Asiatic acid	12.46	neg	533.3486	487, 472, 351	C_30_H_48_O_5_	0.53
40	7,8-Dihydroxy-4-methylcoumarin	1.87	pos	193.0490	119, 95, 147	C_10_H_8_O_4_	-2.82
41	Protocatechuic acid	2.06	pos	155.0333	65, 93, 81, 137	C_7_H_6_O_4_	-2.81
42	Sinapic acid	3.34	pos	207.0646	91, 119, 147	C_11_H_12_O_5_	-2.63
43	Hydroxy-7-methoxycoumarin	3.36	pos	193.0490	193, 178, 133	C_10_H_8_O_4_	-2.58
44	Caffeic acid	3.41	pos	163.0382	89, 117, 135	C_9_H_8_O_4_	-4.09
45	Blumenol A	4.31	pos	207.1372	95, 91, 149	C_13_H_20_O_3_	-3.20
46	*β*-Carboline-1-propionic acid	4.91	pos	241.0981	223, 195, 181	C_14_H_12_N_2_O_2_	4.03
47	3,9-Dihydroxy-4-megastigmene	5.51	pos	213.1842	121, 177, 95	C_13_H_24_O_2_	-3.56
48	Cytisoside 3-O-beta-D-rhamnopyranoside	5.54	pos	593.1836	593, 431, 268	C_28_H_32_O_14_	-4.92
49	4,5-Dehydrovomifoliol	5.79	pos	223.1326	111, 83, 223	C_13_H_18_O_3_	-4.46
50	2,2,6,7-Tetramethylbicyclo[4.3.0]nona-1(9),4-diene-7,8-diol	5.86	pos	209.1526	151, 91, 121	C_13_H_20_O_2_	-3.65
51	2-Adamantanone	5.99	pos	133.1004	77, 115, 133	C_10_H_14_O	-4.90
52	2,2,6,7-Tetramethylbicyclo[4.3.0]nona-1(9),4-dien-8-ol	6.02	pos	175.1474	91, 133, 119	C_13_H_20_O	-3.65
53	Apigenin-7-O-*β*-D-glucoside	6.34	pos	433.1125	271, 433	C_21_H_20_O_10_	-1.01
54	Isolariciresinol 3-alpha-O-beta-D-glucopyranoside	6.41	pos	545.1982	545, 515	C_26_H_34_O_11_	-2.15
55	Carveol	6.59	pos	135.1159	91, 77, 107	C_10_H_16_O	-5.78
56	3,4-Dimethoxycinnamic acid	6.94	pos	209.0803	77, 91, 115	C_11_H_12_O_4_	-2.71
57	Eucommin A	7.03	pos	573.1927	573, 558	C_27_H_34_O_12_	-0.13
58	Schizandriside	7.31	pos	515.1873	515, 440	C_25_H_32_O_10_	-2.95
59	Valerenic acid	7.35	pos	217.1580	79, 77, 133	C_15_H_22_O_2_	-3.04
60	Methoxycoumarin	7.60	pos	177.0537	121, 78, 177	C_10_H_8_O_3_	-5.26
61	Lactucaside	7.88	pos	575.1727	575, 531, 331	C_26_H_32_O_13_	-1.36
62	Genkwanin	8.00	pos	285.0746	285, 242, 270	C_16_H_12_O_5_	-3.96
63	Buddledone A	10.34	pos	203.1785	105, 119, 91	C_15_H_24_O	-4.41
64	Piperine	10.67	pos	286.1426	201, 115, 286	C_17_H_19_NO_3_	-3.98
65	Norpechuelol	11.47	pos	195.1372	79, 77, 81	C_12_H_18_O_2_	-5.64
66	Gardenin B	11.68	pos	359.1117	329, 359, 311	C_19_H_18_O_7_	-2.4
67	7,4′-Dimethoxy-3-hydroxyflavone	11.88	pos	299.0909	299, 256, 284	C_17_H_14_O_5_	-1.8
68	Irone	12.09	pos	224.2001	224, 81, 208	C_14_H_22_O	-4.00
69	Linoelaidic acid	12.48	pos	263.2356	95, 81, 91	C_18_H_32_O_2_	-4.77
70	Oleanonic acid	12.53	pos	455.3503	205, 189, 409	C_30_H_46_O_3_	-3.69
71	2-Palmitoyl-rac-glycerol	12.96	pos	353.2663	67, 151, 277	C_18_H_30_O_3_	-3.40
72	Ursolic acid	14.60	pos	282.2786	457, 203, 189	C_30_H_48_O_3_	-3.60

**Table 2:** COM components and their targets are screened according to the degree of CC-CT network.

Target name	Degree	Compound name	Degree
ESR1	36	Ellagic acid	18
EGFR	36	Pectolinarigenin	14
CDK2	32	Eupatilin	14
GSK3B	26	Acacetin	13
SRC	24	Ursolic acid	12
PPARG	21	Lactucaside	10
CDK1	20	Alangilignoside D	10
IGF1R	19	Abscisic acid	10
INSR	18	Genkwanin	10
ITGAL	16	Luteolin-4′-O-glucoside	10
PIK3CA	12	Rutin	10
ITGB1	12	Isovitexin 2^″^-O-rhamnoside	10
JAK2	11	Hydroxy-7-methoxycoumarin	9
ERBB2	10	7,8-Dihydroxy-4-methylcoumarin	9
HSP90AA1	10	Protocatechualdehyde	9
CDK6	10	7,4′-Dimethoxy-3-hydroxyflavone	9
CDK4	10	Isoorientin 6^″^-O-alpha-L-arabinoside	9
NCOA2	9	Cytisoside 3-O-beta-D-rhamnopyranoside	9
SIRT1	8	Hydroxydihydrobovolide	9
MDM2	8	Eucommin A	8
PIK3R1	8	Calycosin	8
NCOA1	7	Sinapic acid	8
TNNI3	7	Pedunculoside	8
ITGB3	6	Methoxycoumarin	8
NRP1	6	Kaempferol-3-O-rutinoside	8
BCL2L1	6	2,2,6,7-Tetramethylbicyclo[4.3.0]nona-1(9),4-dien-8-ol	7
NFKB1	5	Catechol	7
HIF1A	4	Carveol	7
HDAC2	4	Gardenin B	7
CDKN1A	4	4-Hydroxybenzoic acid propyl ester 4	7
JUN	4	Coumaric acid	7
NFKBIA	4	Scopolin	7
HDAC1	3	Linoelaidic acid	6
EP300	3	Asiatic acid	6
GRB2	2	Sophoricoside	6
CTNNB1	2	Protocatechuic acid	6
PRKAB1	2	Vanillic acid	6
NTRK2	2	Schizandriside	6
PLAUR	2	Isoswertisin 2^″^-O-beta-arabinoside	6
MTOR	2	Chlorogenic acid	6
EZH2	1	Piperine	6
IGF2	1	Salicin	6
SDHB	1	Ethyl p-coumarate	6
IKBKG	1	Ethyl caffeate	6
INS	1	Caffeic acid	6
IRAK1	1	Isochlorogenic acid B	6

**Table 3:** COM components and their targets are screened according to the degree of CC-CT-CP network.

Name	Degree
Ellagic acid	16
Acacetin	12
Ursolic acid	11
Genkwanin	10
Eupatilin	10
Pectolinarigenin	10
7,4′-Dimethoxy-3-hydroxyflavone	9
7,8-Dihydroxy-4-methylcoumarin	9
Alangilignoside D	9
Luteolin-4′-O-glucoside	9
Rutin	9
Protocatechualdehyde	9
Lactucaside	8
Pedunculoside	8
Abscisic acid	8
Cytisoside 3-O-beta-D-rhamnopyranoside	8
Isovitexin 2^″^-O-rhamnoside	8
Hydroxydihydrobovolide	8
Hydroxy-7-methoxycoumarin	8
Isoorientin 6^″^-O-alpha-L-arabinoside	8
Sinapic acid	7
Coumaric acid	7
Gardenin B	7
Eucommin A	7
4-Hydroxybenzoic acid propyl ester4	7
Scopolin	7
Methoxycoumarin	7
Kaempferol-3-O-rutinoside	7
Vanillic acid	6
Protocatechuic acid	6
Catechol	6
Ethyl caffeate	6
Calycosin	6
Ethyl p-coumarate	6
Piperine	6
2,2,6,7-Tetramethylbicyclo[4.3.0]nona-1(9),4-dien-8-ol	6
Schizandriside	6
Carveol	6
Isoswertisin 2^″^-O-beta-arabinoside	6
Salicin	6
Asiatic acid	5
Sophoricoside	5
Sapinofuranone A	5
Linoelaidic acid	5
Isolariciresinol 3-alpha-O-beta-D-glucopyranoside	5
Chlorogenic acid	5
Caffeic acid	5
Cryptochlorogenic acid	5
Isochlorogenic acid B	5
EGFR	45
ESR1	39
GSK3B	37
PIK3CA	34
CDK2	34
SRC	32
PIK3R1	30
IGF1R	28
INSR	27
CDK1	21
PI3K-Akt signaling pathway	21
Thyroid hormone signaling pathway	15
FoxO signaling pathway	13
Focal adhesion	12
HIF-1 signaling pathway	12
Rap1 signaling pathway	11
Ras signaling pathway	10
ErbB signaling pathway	10
Chemokine signaling pathway	9
Neurotrophin signaling pathway	9
Insulin resistance	9
T cell receptor signaling pathway	9
Prolactin signaling pathway	9
Insulin signaling pathway	8
Estrogen signaling pathway	8
B cell receptor signaling pathway	8
Signaling pathways regulating pluripotency of stem cells	7
Toll-like receptor signaling pathway	7
Progesterone-mediated oocyte maturation	7
Adherens junction	7
TNF signaling pathway	6
Adipocytokine signaling pathway	6
p53 signaling pathway	6
Apoptosis	6
Type II diabetes mellitus	5

**Table 4:** Physicochemical properties of COM components.

Compounds	ALog*P*	Molecular solubility	ADMET absorption level
Catechol	1.346	-0.527	0
Vanillic acid	1.201	-1.173	1
Chlorogenic acid	-0.34	-1.8	3
Scopolin	-0.289	-1.69	0
Cryptochlorogenic acid	-0.34	-1.8	3
Isolariciresinol-4-O-Î′-D-glucopyranoside	0.367	-3.006	3
Coumaric acid	1.685	-2.045	0
Ellagic acid	1.584	-0.718	3
Luteolin-4′-O-glucoside	0.238	1.8	3
Isochlorogenic acid B	-0.34	-1.8	3
Sophoricoside	0.21	-2.343	3
Azelaic acid	1.921	-2.061	0
Ethyl caffeate	1.669	-1.869	0
Protocatechuic acid	0.975	-0.703	0
Sinapic acid	1.652	-2.171	0
Caffeic acid	1.443	-1.599	1
Eucommin A	0.237	-3.217	3

**Table 5:** Potential target information filtered by network topology parameters.

Target	DC	NC	CC	LAC	BC	EC
CDK1	5	1.5	0.012017937	0.8	244.46666	0.031225344
CDK4	5	0.5	0.012112082	0.4	498.20758	0.0964223
NFKBIA	5	3.583333	0.012027286	2	1029.3334	0.020313684
TP53	13	3.469048	0.01216707	1.230769	2694.152	0.28619927
CDK6	4	0.666667	0.012051805	0.5	22.145056	0.041681297
EP300	16	5.057937	0.012175915	1.625	3737.9062	0.36668986
PTPN1	3	1	0.012096047	0.666667	240.25555	0.034410365
CDKN1A	8	4.547619	0.012100415	2	815.6494	0.109220006
JUN	8	0.571429	0.012144282	0.5	1198.7222	0.14934167
CCNA2	3	2	0.012017937	1.333333	2.3333333	0.03635606
AR	5	1	0.012147217	0.8	272.68796	0.1654807
HDAC2	4	1.5	0.012109163	1	303.5	0.09623724
AKT1	3	1.5	0.012104059	0.666667	936	0.06611888
PIK3R1	6	0.4	0.012146483	0.333333	1201.9866	0.09323144
NCOA1	9	0.708333	0.012138414	0.444444	868.9675	0.17657834
PPARG	4	1.333333	0.012102601	1	116.26695	0.11342621
STAT3	7	1.433333	0.012166333	1.142857	990.3526	0.2125998
RELA	6	1.116667	0.012104788	0.666667	2047.7476	0.09581511
HIF1A	6	3	0.012153828	2	415.76923	0.2106278
DNMT1	3	2.5	0.012072797	1.333333	7.6666665	0.051221147
HDAC1	7	3.833333	0.012151623	1.714286	597.0825	0.20369877
CTNNB1	10	0.906746	0.012164125	0.6	1957.5703	0.22608843
EGFR	6	1.1	0.012161917	0.666667	1579.914	0.1445954
NFKB1	4	2.166667	0.012025127	1.5	343.33334	0.01961755
SIRT1	3	1	0.012115002	0.666667	240	0.1012076
CDK2	8	1.964286	0.012099687	1	612.7579	0.098931395
CCNE1	3	2	0.012017937	1.333333	2.3333333	0.03635606
HSP90AA1	20	3.419445	0.012185511	0.7	4693.081	0.3599591
CCND1	5	1.583333	0.01212304	1.2	573.6047	0.09457812
ESR1	10	1.328968	0.012189945	1	3201.1619	0.27561933
GRB2	11	0.7	0.012121578	0.181818	2374.0754	0.0761091

**Table 6:** The BE of molecular docking between the bioactive components and the core predicted targets.

Ligand	Proteins	Affinity (kcal/mol)	Ligand	Proteins	Affinity (kcal/mol)
7,4′-Dimethoxy-3-hydroxyflavone	ESR1	-8.4	Coumaric acid	ESR1	-6.5
	EGFR	-8.4		EGFR	-6.4
	CDK2	-7.1		CDK2	-6.2
	CDK1	-7.3		CDK1	-5.9

Abscisic acid	ESR1	-8.2	Sinapic acid	ESR1	-6.3
	EGFR	-6.8		EGFR	-6.2
	CDK2	-6.9		CDK2	-6.1
	CDK1	-6.6		CDK1	-6.3

Acacetin	ESR1	-8.1	Isochlorogenic acid B	ESR1	-6.2
	EGFR	-8.5		EGFR	-9.4
	CDK2	-7.1		CDK2	-3.9
	CDK1	-7.6		CDK1	-7.3

Genkwanin	ESR1	-8.1	Protocatechuic acid	ESR1	-6.2
	EGFR	-8.3		EGFR	-5.9
	CDK2	-7.2		CDK2	-5.5
	CDK1	-7.7		CDK1	-5.7

Chlorogenic acid	ESR1	-7.6	Hydroxydihydrobovolide	ESR1	-6.1
	EGFR	-8.3		EGFR	-6.2
	CDK2	-7.4		CDK2	-5.9
	CDK1	-7.3		CDK1	-5.6

Calycosin	ESR1	-7.5	Vanillic acid	ESR1	-6.1
	EGFR	-8.5		EGFR	-5.4
	CDK2	-7.0		CDK2	-5.6
	CDK1	-7.4		CDK1	-5.8

Piperine	ESR1	-7.4	Carveol	ESR1	-6
	EGFR	-8.8		EGFR	-5.3
	CDK2	1.5		CDK2	-5.9
	CDK1	-7.4		CDK1	-5.7

Scopolin	ESR1	-7.4	4-Hydroxybenzoic acid propyl ester	ESR1	-5.8
	EGFR	-7.5		EGFR	-5.8
	CDK2	-6.6		CDK2	-5.9
	CDK1	-7.2		CDK1	-5.7

Alangilignoside D	ESR1	-7.2	Protocatechualdehyde	ESR1	-5.8
	EGFR	-8.4		EGFR	-5.8
	CDK2	-2.0		CDK2	-5.2
	CDK1	-5.6		CDK1	-5.3

Ethyl caffeate	ESR1	-7.2	Catechol	ESR1	-5.3
	EGFR	-6.9		EGFR	-5.1
	CDK2	-6.3		CDK2	-4.8
	CDK1	-6.7		CDK1	-4.7

Eupatilin	ESR1	-7.2	Schizandriside	ESR1	-4.3
	EGFR	-8.0		EGFR	-8.2
	CDK2	-5.0		CDK2	1.2
	CDK1	-7.0		CDK1	-6.8

Pectolinarigenin	ESR1	-7.2	Sophoricoside	ESR1	-4
	EGFR	-8.3		EGFR	-9.8
	CDK2	-5.0		CDK2	—
	CDK1	-7.1		CDK1	-7.1

Ellagic acid	ESR1	-7.0	Kaempferol-3-O-rutinoside	ESR1	-2.5
	EGFR	-8.8		EGFR	-7.3
	CDK2	-8.8		CDK2	5.6
	CDK1	-8.8		CDK1	-3.7

Methoxycoumarin	ESR1	-7.0	Isolariciresinol 3-alpha-O-beta-D-glucopyranoside	ESR1	-1.7
	EGFR	-6.6		EGFR	-8.7
	CDK2	-6.6		CDK2	-2.5
	CDK1	-6.5		CDK1	-5.9

7,8-Dihydroxy-4-methylcoumarin	ESR1	-6.9	Rutin	ESR1	-0.2
	EGFR	-6.8		EGFR	-8.9
	CDK2	-7.3		CDK2	5.1
	CDK1	-7.1		CDK1	-5.2

Ethyl p-coumarate	ESR1	-6.9	Lactucaside	ESR1	0.4
	EGFR	-6.3		EGFR	-9.1
	CDK2	-6.3		CDK2	11.1
	CDK1	-6.1		CDK1	-5.6

Hydroxy-7-methoxycoumarin	ESR1	-6.8	Isoswertisin 2^″^-O-beta-arabinoside	ESR1	1.3
	EGFR	-7.3		EGFR	-7.1
	CDK2	-7.0		CDK2	5.1
	CDK1	-6.5		CDK1	-4.8

Salicin	ESR1	-6.8	Cytisoside 3-O-beta-D-rhamnopyranoside	ESR1	1.6
	EGFR	-6.4		EGFR	-7.6
	CDK2	-7.0		CDK2	7.2
	CDK1	-6.6		CDK1	-2.9

Linoelaidic acid	ESR1	-6.7	Isoorientin 6^″^-O-alpha-L-arabinoside	ESR1	1.8
	EGFR	-6.6		EGFR	-8.9
	CDK2	-5.9		CDK2	—
	CDK1	-5.9		CDK1	3.6

Gardenin B	ESR1	-6.6	Ursolic acid	ESR1	1.8
	EGFR	-8.3		EGFR	-7.6
	CDK2	-5.1		CDK2	—
	CDK1	-7.8		CDK1	-3.9

Caffeic acid	ESR1	-6.5	Luteolin-4′-O-glucoside	ESR1	4
	EGFR	-6.8		EGFR	-8.1
	CDK2	-6.0		CDK2	—
	CDK1	-6.0		CDK1	-7.4

Asiatic acid	ESR1	4.4	Pedunculoside	ESR1	5.6
	EGFR	-8		EGFR	-5.6
	CDK2	11.6		CDK2	36.4
	CDK1	-2.4		CDK1	0.1

Isovitexin 2^″^-O-rhamnoside	ESR1	6.9	Eucommin A	ESR1	17.7
	EGFR	-8		EGFR	-7.2
	CDK2	—		CDK2	—

**Table 7:** Results of molecular docking between the main Q-marker and the core predicted targets.

Ligand	Proteins	Affinity (kcal/mol)	Residues	Hydrogen bonds	Pi interactions
Caffeic acid	ESR1	-6.5	LEU346, LEU349, ALA350, LEU387, MET388, LEU391, ARG394, LEU428, PHE425	PHE404, GLU353 (3.0 A)	PHE404 (3.0 A)
	EGFR	-6.8	VAL726, ILE789, THR790, LEU788, THR854, CYS775, ASP855, ARG776, LEU777, MET776	LYS745, ALA743 (3.0 A)	—
	CDK2	-6.0	ASN132, ILE10, VAL18, ALA31	—	—
	CDK1	-6.0	MET785, LEU83, LEU135, GLN132, ILE10, VAL18, LYS33	ASP86 (3.0 A)	—

Chlorogenic acid	ESR1	-7.6	LEU349, GLU353, ALA350, LEU346, LEU525, HIS524, GLY521, MET421, TRP383, LEU384, MET388, LEU391, ARG394, ILE424, PHE425	—	PHE404 (3.0 A)
	EGFR	-8.3	GLY796, LEU1001, LEU718, GLY719, PHE723, LEU792, GLY721, VAL726, ALA722, LYS745	MET793, GLY724 (3.0 A)	—
	CDK2	-7.4	GLN65, ASP86, LEU134, GLN131, VAL64, ALA144, ASP145, LYS33, PHE82, ILE10, ALA31, PHE80, GLY11, VAL18	LEU83 (3.0 A)	—
	CDK1	-7.3	SER84, MET85, LEU83, ASP86, GLU12, LYS33, LEU135, PHE82, GLN132, ASN132, GLY13, ALA145, GLU8, LYS20, ILE10, ALA31, LYS9, VAL18	—	LYS89 (4.0)

Ellagic acid	ESR1	-7.0	LEU525, HIS524, GLY521, MET421, ILE424, MET388, LEU391, LEU346, ALA350, THR347, LEU428	—	PHE404 (3.0 A)
	EGFR	-8.8	LEU1001, LEU718, VAL726, LYS745, ALA743, THR790, GLY796, LEU792, MET793, LEU844	ASP855 (3.0 A)	—
	CDK2	-8.8	PHE82, GLN81, LEU134, VAL64, LYS33, VAL18, VAL31, ILE10	ASP145, LEU83 (3.0 A)	—
	CDK1	-8.8	PHE82, LEU135, ASP86, PHE80, ALA31, ILE10, VAL18, GLY11, GLY13, GLU12	LYS33, LEU83 (3.0 A)	—

Vanillic acid	ESR1	-6.1	LEU349, ALA350, GLU353, LEU346, PHE404, LEU384, LEU387, MET388, LEU391, ARG394	—	—
	EGFR	-5.4	LEU718, VAL726, LYS745, ASP855, THR854, LEU844, LEU792	MET793 (3.0 A)	—
	CDK2	-5.6	ASN132, ASP145, ALA144, LEU134, VAL64, PHE88, PHE82, LEU83, ALA31, ILE10	GLU81 (3.0 A)	—
	CDK1	-5.8	PHE82, GLU81, LEU135, ASP86, GLN132, ILE10, VAL18, LYS33, ALA31	LEU83 (3.0 A)	—

## Data Availability

The data used to support the findings of this study are included within the article.
